# Home Care Services for Seniors: A Typology of Instruments and Administrative Burdens in Industrialized Countries

**DOI:** 10.1093/geroni/igab046.934

**Published:** 2021-12-17

**Authors:** Patrik Marier, Daniel Dickson, Kyuho Lee

**Affiliations:** 1 Concordia University, Montreal, Quebec, Canada; 2 Daegu University, Daegu University, Kyongsang-bukto, Republic of Korea

## Abstract

This contribution has two key objectives. First, inspired by earlier studies in comparative welfare state and in (social) gerontology, we develop a conceptualization of autonomy that is rooted in its social dimensions. This concept is then deployed to assess its policy considerations within the field of home care, both with regards to access and generosity in 21 industrialized countries. Second, this contribution performs a comparative assessment of the key factors resulting in a prioritization of the social dimensions of home care and social services in long term care. This study involves an-depth analysis of policy instruments deployed by public authorities to enhance the (social) autonomy of older adults, complemented with interviews with policy makers in diverse home care policy settings (Canada, France, South Korea, Sweden, and the United States). As such, this study features an evaluation of the presence of social elements in the definition and supply of care needs across 21 countries. It leads to the construct of a social dimensions of autonomy index based upon these instruments and the budgetary prioritization of home care within long term care policies. Among core findings, one discovers broader access and more generous funding when home care responsibilities are firmly embedded at the local level.

